# Sensorimotor Skills Impact on Temporal Expectation: Evidence from Swimmers

**DOI:** 10.3389/fpsyg.2017.01714

**Published:** 2017-10-04

**Authors:** Marco Bove, Laura Strassera, Emanuela Faelli, Monica Biggio, Ambra Bisio, Laura Avanzino, Piero Ruggeri

**Affiliations:** Department of Experimental Medicine, Section of Human Physiology, University of Genoa, Genoa, Italy

**Keywords:** temporal expectation, timing, swimming, sport, sensorimotor skills, temporal occlusion

## Abstract

Aim of this study was to assess whether the ability to predict the temporal outcome of a sport action was influenced by the sensorimotor skills previously acquired during a specific sport training. Four groups, each of 30 subjects, were enrolled in this study; subjects of three groups practiced different sports disciplines (i.e., swimming, rhythmic gymnastics, and water polo) at competitive level whilst the fourth group consisted of control subjects. Subjects were asked to observe a video showing a swimmer doing two laps in crawl style. This video was shown 36 times, and was occluded after variable intervals, randomized across trials, by a dark window that started 3, 6, and 12 s before the swimmer touched the poolside. During the occluded interval, subjects were asked to indicate when the swimmer touched the edge of the pool by clicking on any button of the laptop keyboard. We found that swimmers were more accurate than subjects performing other sports in temporally predicting the final outcome of the swimming task. Particularly, we observed a significant difference in absolute timing error that was lower in swimmers compared to other groups when they were asked to make a temporal prediction with the occluded interval of short duration (i.e., 3 s). Our findings demonstrate that the ability to extract temporal patterns of a motor action depends largely on the subjective expertise, suggesting that sport-acquired sensorimotor skills impact on the temporal representation of the previously observed action, allowing subjects to predict the time course of the action in absence of visual information.

## Introduction

Timing is a fundamental feature of human movement, perception, and cognition (Avanzino et al., 2015a). In action, timing is crucial when producing movement sequences and when coordinating our movements to those of various moving objects in the external environment (Ivry and Spencer, 2004). Also, the processing of time-dependent features of movement has a crucial role in predicting whether the outcome of a complex motor sequence is consistent with its ultimate goal.

In daily life we continuously observe people moving around us and, implicitly, we are able to perceive and extrapolate several information concerning the spatio-temporal features of their movements. By extracting temporal patterns and regularity of events, people are capable to predict how the movements evolve over time. This ability is referred as temporal expectation (Nobre et al., 2007). The temporal expectation of events is often discussed in terms of ‘hazard functions,’ i.e., the conditional probability of an event occurring at a given time given that it has not yet occurred (Luce Duncan, 1986; Nobre et al., 2007). In elite athletes the ‘hazards of time’ can be very risky and cause unpleasant consequences; as in the summer of 2012, when the American swimming team won the 4×100 medley relay at FINA Barcelona’s world swimming championship but it was excluded because of the first irregular change. In swimming – relay events, the time prediction is fundamental for the success of the change. In this crucial moment, the swimmer on the block has to expect the exact moment when the partner touches the edge of the pool to leave the block. In the last meters of swimming of his/her teammate, before preparing itself to plunge, the swimmer at the starting block does not watch the teammate but makes a prediction of when the partner will touch the edge and gets ready to detach the feet in that instant. Even if an optimal temporal expectation is a feature necessary to be an elite athlete, to the best of our knowledge, there are only few works in the literature investigating this topic in sport (Tobin and Grondin, 2012). In particular, whether the acquired motor skills can influence the ability to predict the temporal consequences of a motor act is an aspect that needs to be deeply investigated in sport domain.

There is a large body of literature showing that action and perception share common neurophysiological substrates (Rizzolatti and Craighero, 2004) and common representational codes (Hommel et al., 2001; Schutz-Bosbach and Prinz, 2007). As a consequence, it was suggested that motor repertoire and perceptual representation may prime each other, as shown in studies on motor learning through observation (Vogt, 1995; Mattar and Gribble, 2005) and in paradigms involving action prediction through internal simulation (Schutz-Bosbach and Prinz, 2007; Springer et al., 2013). To this propose, the occlusion technique was used in several works investigating how motor repertoire affects action prediction. Participants were asked to observe familiar actions that were briefly occluded from view. Typically, the task for the participants was to judge whether the part of the action appearing after the occlusion was an accurate continuation of that observed before occlusion or not (Graf et al., 2007; Springer et al., 2011; Parkinson et al., 2012). A modified version of this paradigm was adopted by Avanzino et al. (2013) and Martino et al. (2015) to assess temporal expectation abilities in dystonic patients. During this task subjects were asked to predict the end of the motion, performed by a human and by a non-biological stimulus, when the final part of the movement was completely occluded, after a variable interval from its onset, by a dark screen of variable duration.

These studies rely on the hypothesis that a sort of “action simulation,” namely the activation of cortical and subcortical mechanisms similar to those involved in movement execution, occurs during action observation, and remains active to help the participant to reconstruct the missing part of the observed motion (Jeannerod, 2001, 2006). Therefore, if internal simulation involves motor resources, one would expect that people who developed a specific expertise in motor domains show a special perceptual sensitivity to actions that fall in their expertise. Strong support for this hypothesis comes from the studies on a population of expert dancers showing that brain regions involved in movement execution are more strongly engaged when the dancer watched her/his own dance style (already represented in the motor repertoire) compared to other styles in which they have no previous motor experience (Calvo-Merino et al., 2005, 2006). This “perceptual resonance” (Schutz-Bosbach and Prinz, 2007) pertains also the temporal features of a movement. Indeed, the link between executed and perceived tempo has been established in works showing that during action observation the perception of the temporal properties of a movement is modulated by one’s own internal movement tempo, which, in turn, influences the motor system’s activity and response (Gavazzi et al., 2013; Avanzino et al., 2015b).

The ability to represent temporal patterns of motor actions was examined in sport, dance, and music. Based on temporal parameters, Murgia et al. (2012) showed that expert golfers recognized as own movement a motion that produced a sound characterized by temporal features that mirrored their own. Tap dancing experience was shown to be crucial for detecting temporal deviations in tap dance performance recordings (Murgia et al., 2017) and musicians were found to be better in detecting temporal deviations in their own performances than in other musicians’ performances (Repp and Keller, 2010). Overall, these studies suggest that gaining a sensorimotor experience in a specific motor domain contributes to the development of a temporal representation of complex movements.

The aim of the present study was to test whether the ability to predict the temporal outcome of a sport action (here a swimming-relay event) was influenced by the sensorimotor skills previously acquired by the subjects during specific sport training. To this purpose, we enrolled four groups of subjects with different motor expertise. Three groups were composed of athletes playing different sports: swimming, water polo, and rhythmic gymnastics. The swimmers were chosen because their motor expertise was specific for the experimental task, whilst water-polo and rhythmic gymnastics were included because of their good knowledge of the motor task and their good timing abilities, respectively. The control group was composed of subjects who did not played sport at competitive level. We administered a modified version of the paradigm proposed by Avanzino et al. (2013) and Martino et al. (2015) aiming to investigate the ability to temporally predict the end of a visually perceived free style swimming task as it occurs in swimming-relay events, and, in particular, to predict the exact time when the swimmer at the end of the task touched the edge of the pool. We hypothesized that swimmers would be more accurate than the other groups in predicting the temporal outcome of this swimming task.

## Materials and Methods

### Participants

A total of 120 subjects, thirty swimmers (12 males and 18 females, mean age ± standard deviation (*SD*) = 15 ± 2 years), thirty water polo players [15 males and 15 females, mean age ± (*SD*) = 14 ± 1.7 years], thirty rhythmic gymnasts [30 females, mean age ±*SD* = 14 ± 2 years] and thirty control subjects [13 males and 17 females, mean age ±*SD* = 16 ± 1.2 years] were recruited. All participants had a normal or corrected-to-normal vision and none had a previous history of neuromuscular or neurological disorder. The groups of swimmers, water polo players, and rhythmic gymnasts were composed by competitive athletes (regional and national levels) (see **Table 1** for details). Control subjects were selected by the school sport science professor, and subjects with coordination disorders were excluded from the study. Control participants played sport (volleyball, soccer, athletics, basketball, dance, equitation, and karate) only at recreational level (no more than 1 h per week outside school, plus 2 h at school). None of them was involved in swim, water polo, or rhythmic gymnast. During the experiment, participants from all groups were involved in their regular sport activities. A parental written informed consent was required prior to participation in the study. The study protocol was approved by the ethics committee of the University of Genoa according to the 2013 revision of the Declaration of Helsinki on human experimentation.

**Table 1:** Characteristics of competitive athletes. Values are expressed as mean ± standard deviations.

	Years of practice	Hours of training per week in the last year
Swimmers	9.96 ± 2.79	10.73 ± 1.62
	range: 5–15 years	range: 6–12 h
Water polo players	5.88 ± 7.29	10.66 ± 2.22
	range: 4–10 years	range: 6–15 h
Rhythmic gymnasts	7.17 ± 2.72	13.03 ± 7.49
	range: 3–13 years	range: 6–27 h

### Experimental Design

**FIGURE 1:**
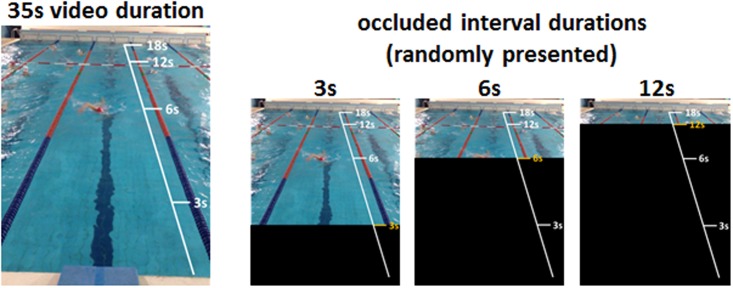
Schematic view of the experimental design. A video that lasted 35 s showing a swimmer performing two laps of front crawl at a fixed speed, from the perspective of the participant on the starting block, was shown to the subjects (Left). The video was occluded, after a variable interval from its onset, by dark windows of variable duration (3, 6, or 12 s) (Right). During the occluded interval, participants were required to extrapolate the temporal duration of the displayed movement by clicking on any button of the keyboard when they thought the movement had reached its end.

The task is a modified version of that designed by our group (Avanzino et al., 2013; Martino et al., 2015) and exploits the temporal occlusion technique (Jones and Miles, 1978; Murgia et al., 2014; Sors et al., 2017). Participants were seated on a comfortable chair in a quiet room. They were requested to look at a computer screen where a video of a professional swimmer was displayed. The video (total duration = 35 s) showed, from the participant’s perspective on the starting block, a swimmer doing two laps of frontal crawl executed at a constant pacing rate. The video ended when the swimmer touched the edge of the swimming pool. The task consisted of observing the video that was occluded, after a variable interval from its onset (pre-occluded interval), by a black screen of variable duration (occluded interval). Three different occluded interval durations, randomized across trials, were used. The occluded interval started 3, 6, or 12 s before the end of the video. Each condition was administered 12 times in a randomized order, for 36 trials per experimental session. During the occluded interval, subjects were required to indicate, by clicking on any button of the keyboard, when the movement reached its end, namely when they thought the swimmer touched the edge of the swimming pool. Once they pressed the key, the black screen disappeared. Participants did not receive any feedback on the occluded interval duration or on their performance throughout the whole session. They were just given the chance to watch the whole performance once before starting the session. Instructions to the participants were written in a white window preceding the onset of the video; no further assistance was provided. The tasks have been programmed using dedicated software (E-Prime 2.0, SciencePlus). The experimental paradigm is summarized in **Figure 1**.

### Data Analysis

#### Data Treatment

Performance on the temporal expectation task was evaluated by means of the absolute values of timing error, the percentage of anticipation responses and the coefficient of variability. The timing error was calculated as the difference between the duration of the occluded interval (occluded interval duration: 3, 6, or 12 s) and the period spanning from the beginning of the occluded interval to the subject’s response (reproduced interval). The absolute value of this error was then normalized with respect to the corresponding occluded interval duration and was expressed as a percen-tage ([abs(occluded interval duration - reproduced duration)/occluded interval duration]×100); only normalized absolute timing errors were analyzed. This parameter provides a direct measure of the accuracy of subjects in estimating the corresponding occluded interval: the greater the absolute timing error, the greater the error in temporal judgment regardless of its direction. The percentage of responses in anticipation (calculated on the total number of responses) represents the number of responses in which subjects pressed the space button before the real end of the movement. This parameter gave information on the tendency to under- or over-estimate the target interval. Finally, the coefficient of variation was calculated as SD/mean of the reproduced intervals, representing an index of performance variability in temporal estimation.

### Statistical Analysis

Timing parameters (absolute timing error, percentage of responses in anticipation and coefficient of variation) were analyzed by means of a repeated measures ANOVA with GROUP (swimmers, rhythmic gymnasts, water polo players, and control participants) as between-subjects factor and OCCLUDED INTERVAL (3, 6, and 12 s) as within-subjects factor. *Post hoc* analysis was performed by means of the Newman–Keuls test. The probability level taken to indicate the significance was *p*-value < 0.05.

## Results

**FIGURE 2:**
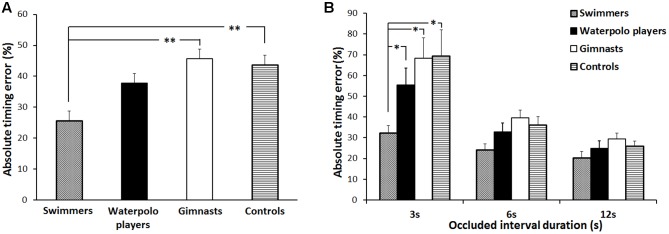
Absolute timing error. **(A)** Mean values over the three occluded interval durations associated to the four groups of subjects. **(B)** Mean values associated to the four groups of subjects and to the three occluded interval durations (3, 6, and 12 s). Error bars refer to the standard error of the mean. ^∗^*p* < 0.05 and ^∗∗^*p* < 0.01.

The result of the statistical analysis on the absolute timing error showed a significant main effect of GROUP [*F*(3,116) = 3.61, *p* = 0.02, 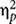 = 0.09] (**Figure 2A**). *Post hoc* analysis indicated that, irrespective of the duration of the occluded interval, swimmers exhibited a significantly lower absolute timing error with respect to the rhythmic gymnasts (*p* = 0.003) and control subjects (*p* = 0.008). Noteworthy, by averaging the timing error related to the three occluded intervals, swimmers did not differ from the water polo players (*p* = 0.07). The performances of the non-swimmer groups were always comparable.

However, statistical analysis showed also that when the duration of the occluded interval was 3 s swimmers exhibited a lower absolute timing errors compared with all the other groups.

Accordingly, a significant OCCLUDED INTERVAL^∗^GROUP interaction [*F*(6,232) = 2.66, *p* = 0.02, 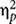 = 0.06] was found. *Post hoc* revealed that the absolute timing error for swimmers was significantly lower than those of water polo players (*p* = 0.04), rhythmic gymnasts (*p* = 0.003) and control subjects (*p* = 0.002) when the occluded interval duration was 3 s. No significant differences appeared among the swimmers and the other groups in correspondence of the 6 and 12 s occluded interval durations. Further, the absolute timing error for swimmers was not modulated by the duration of the occluded interval. Conversely, in the other groups the percentage error made in correspondence to the 3 s-occluded interval was significantly higher than those made at the 6 and 12 s-occluded intervals (rhythmic gymnasts, always *p* < 0.001; water polo players, always *p* < 0.001; control subjects, always *p* < 0.001) (**Figure 2B**).

**FIGURE 3:**
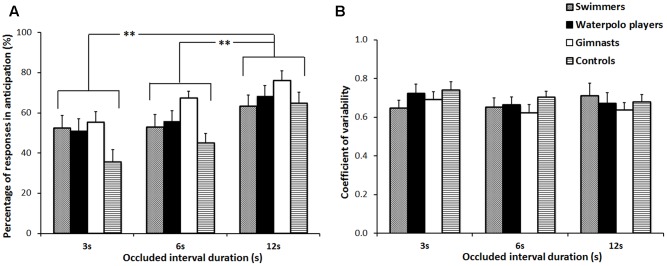
Percentage of responses in anticipation **(A)** and coefficient of variability **(B)**. Mean values over the three occluded interval durations (3, 6, and 12 s) associated to the four groups of subjects. Error bars refer to the standard error of the mean. ^∗∗^*p* < 0.01.

The analysis on the percentage of responses in anticipation showed a significant main effect of the factor OCCLUDED INTERVAL [*F*(2,232) = 30.48, *p* < 0.001, 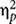 = 0.21], indicating an increment with increasing occluded interval duration (3 vs. 6, *p* = 0.009; 3 vs. 12, *p* < 0.001; 6 vs. 12, *p* < 0.001) (**Figure 3A**). No differences among groups were found as well as not significant interaction. At last, no significant effects were found in the analysis of the coefficient of variability (**Figure 3B**).

## Discussion

In the present study we showed that swimmers were more accurate than subjects performing other sports in temporally predicting the final outcome of a swimming task. Particularly, the main difference between swimmers and the other groups involved in this study was found when they were asked to make a temporal prediction with the occluded interval of short duration (i.e., 3 s). This time interval matches with the time during which, in a relay event, the swimmer on the starting block does not watch the teammate swimming toward the pool side but makes a prediction of when the partner will touch the edge. Conversely, no difference in temporal prediction was observed between swimmers and the other groups for the occluded intervals of larger duration (i.e., 6 and 12 s). Also, the temporal error analysis showed that, by averaging the timing error related to the three occluded intervals, water polo players did not differ from the swimmers. Further, the normalized absolute timing error for swimmers was not modulated by the duration of the occluded interval, unlike in the other groups, whose ability to predict the end of motion was influenced by the occluded interval duration.

Our findings point out that the ability to extract temporal patterns of a sport motor action is strongly influenced by the subjective expertise, suggesting that sport-acquired sensorimotor skills in specific context conditions strongly affect the temporal representation of action. Indeed, although water polo players have a good knowledge of the specific motor task and a generalized ability in temporally predicting swimming actions, they were less accurate than swimmers only in that temporal condition referring to a task specifically trained in the swimmers such as the swimming relay event. On the other hand, we showed that although the movement of a rhythmic gymnastics routine involves a high degree of athletic skill and a high ability in perceiving *tempo* this is not enough to distinguish the group of the rhythmic gymnasts in this specific motor task. All these findings are in line with previous works showing that the ability of the athletes to acquire an excellent aptitude in perceiving and performing fast and complex actions in the space–time domain is strongly related to the sport training program (Bootsma et al., 1990; Aglioti et al., 2008), during which athletes gain a comprehensive experience of the trained movement. Indeed, the ability to represent temporal patterns of motor actions can refer to various sensory domains. For example, it has been shown that the ability to reproduce the duration of a moving stimulus depends largely on the spontaneous movement tempo and, crucially, on the possibility to match the internal motor repertoire with the observed kinematics (Gavazzi et al., 2013). In sport, a recent study applying a temporal occlusion paradigm showed that skilled with respect to less-skilled goalkeepers were more accurate in predicting the direction of the penalty kick shown in the observed video, suggesting that motor expertise is involved in reconstructing the missing part of an observed action (Causer et al., 2017). Furthermore, also acoustic information dealing with sport sounds are integrated by athletes and associated with a specific sport action (Murgia et al., 2012). These and other studies (Repp and Keller, 2010; Murgia et al., 2017) suggested that obtaining a sensorimotor experience in a specific multisensory domain strongly influences the development of the temporal representation of a complex movements.

Another aspect that needs to be taken into account is the possibility that during occlusion participants simulated the previously observed swimming action. The mental simulation of action is a phenomenon that can be observed also in children starting from the age of 5 and improved from 10 years of age (Spruijt et al., 2015). Recently, it has been shown that motor imagery can be performed also during action observation (Eaves et al., 2016). One can speculate that the motor imagery processes engaged during action observation are maintained also when the observed action is occluded. Therefore, we could suggest that during action observation swimmers might have started motor imagery processes, and that, being the temporal congruence between the observed and the imagined action higher than those of the other group, they were able to make a better temporal prediction. However, the ability to maintain the temporal congruency presumably faded over time, and this might be a possible mechanism explaining the lack of group differences at longer occlusion points.

It has been demonstrated that in adult experienced swimmers knowledge of the task duration improved temporal performance across different temporal tasks (time estimation and production) and duration ranges, suggesting that task duration knowledge is strongly involved in time perception (Tobin and Grondin, 2012). Recently the same group showed that expert runners were more accurate than intermediate runners for both predicting and estimating their running time (Tobin and Grondin, 2015). All these studies focused on explicit timing mechanisms and, to our knowledge it has not been investigated whether the acquired motor skills can influence implicit timing mechanisms. The crucial distinction between explicit and implicit timing is whether or not the task instructions require subjects to provide an overt estimate of duration (Coull and Nobre, 2008). In motor or perceptual tasks of explicit timing, the ‘task goal’ for the subject is to provide an accurate estimate of the elapsed time. Implicit timing, on the contrary, is engaged as a by-product of non-temporal task goals, when sensory stimuli or motor responses nevertheless adhere to a strict temporal framework (Coull and Nobre, 2008). For example, task instructions may require subjects to make a perceptual judgment about stimulus features or to perform a specific motor act. Our results showed that the sensorimotor skills acquired during an extensive training (such as the athletes do with their sport) exerted an influence also in implicit timing mechanisms.

Notably implicit timing tasks, when timing information is processed to make predictions on the outcome of a motor act, are likely to engage the same cerebral network usually engaged in action selection, including cerebellum. Indeed, we showed that inhibiting the activity of the lateral cerebellum with 1 Hz-repetitive transcranial magnetic stimulation (TMS) disrupted time estimation of a human motor act, rather than that of a moving object (not a biological motion), suggesting that cognitive and perceptual functions of cerebellum are likely to be strictly connected to motor control (Avanzino et al., 2015a). In agreement with the role of cerebellum in processing and adapting motor related information, it has been demonstrated that the cerebellum is needed to optimize self-action by recalibrating predictions capturing the sensory consequences of one’s actions (Bell et al., 2008; Synofzik et al., 2008; Izawa et al., 2012).

Further, in a closed skill sport like swimming, the alternate and rhythmic repetition of upper limb movements during freestyle can improve temporal expectation. Indeed, regular, rhythmic stimulation has been shown to induce strong temporal expectation, facilitating processing of events occurring at the predicted beat (Schroeder and Lakatos, 2009; Jones, 2010; Mathewson et al., 2010). Temporal expectation, when induced by rhythmic patterns, could directly modulate perceptual excitability and influence early visual psychophysical functions. Lakatos et al. (2008) and Schroeder and Lakatos (2009) suggested that when the brain can detect a rhythm in a task, attention enforces phase resetting and entrainment of neuronal excitability oscillations to the relevant stimulus stream, thus changing response gain and amplifying neuronal responses to the events in that stream (Rohenkohl et al., 2012).

Therefore, we can speculate that the acquired motor experience in swimming might allow the swimmers group to better recognize the rhythmic aspects of the observed movements and, thus, to amplify the neuronal responses in those areas where the action outcome is simulated during the 3 s-occluded interval. Recently, it has been demonstrated that the left dorsal premotor cortex (PMd) might contribute to predicting action in the absence of visual information (Stadler et al., 2012), but only at the moment when an observed action becomes occluded and may hand over to other regions when ongoing prediction has to be maintained. This latter evidence might explain the lack of differences between swimmers and the other groups with occluded intervals larger than 3 s. In fact, we might hypothesize that neural structures involved in simulating the observed actions, such as the left PMd, can optimally work as action predictor in absence of visual information in a range of seconds but their predictive ability is reduced with time. Thus, in case of large temporal intervals, the temporal prediction task is passed to other neural structures that likely are less influenced by motor experiences.

A noteworthy aspect of our study is that implicit timing mechanisms have been studied in a sample of young agonistic swimmers with a regional experience of competition. Our results might suggest that in the early learning phase of the swimming styles the information concerning the temporal aspects of movement are rapidly stored and consolidated during the daily training. In agreement with this idea, it has been demonstrated that in young tennis players the timing accuracy improves mainly between the ages of 7 and 10 and that the tennis practice accelerates the development of timing accuracy (Benguigui and Ripoll, 1998). Also, Rattat and Droit-Volet (2007) investigated young children’s ability to maintain in long-term memory a duration of an action they have previously experienced (i.e., implicit long-term memory for duration) showing that even after a 6-month delay, children were still better than baseline (i.e., their performance level before the training session).

In swimmers, the percentage of responses given in anticipation with respect to the end of the motion changed as a function of the duration of the occluded interval as in the other three groups. In particular, in all groups we observed that longer occluded intervals were associated with higher tendency to shift the end of the perceived movement ahead in time, as already found in previous studies using the same experimental paradigm (Avanzino et al., 2013; Martino et al., 2015). Indeed, when humans are asked to reproduce various temporal intervals, longer durations are perceived as being shorter than the reference, and the opposite is true for short durations, i.e., the Vierordt’s law (Vierordt, 1868), and it has also been found in studies of ‘pure’ temporal reproduction (Bangert et al., 2011).

## Conclusion

This study shows that the ability to implicitly predict the temporal outcome of a motor action is significantly influenced by the subjective expertise. This suggests that sensorimotor skills acquired during specific motor training affect the temporal representation of a previously observed action, allowing subjects to predict the time course of the action in absence of visual information. Implicit motor imagery mechanisms might be involved in this process likely facilitated by prior sensorimotor experiences.

## Author Contributions

Conception of the work: MBo, LS, LA, and PR. Design of the work: MBo, LS, MBi, LA, and PR. Acquisition of the data: LS and EF. Analysis and interpretation of data: MBo, MBi, AB, PR, and EF. Drafted the work: MBo, LS, AB, and PR. Revised the work: EF, MBi, AB, and LA. Final approval of the version to be published: MBo, LS, EF, MBi, AB, LA, and PR. Agreement to be accountable for all aspects of the work in ensuring that questions related to the accuracy or integrity of any part of the work are appropriately investigated and resolved: MBo, LS, EF, MBi, AB, LA, and PR.

## Conflict of Interest Statement

The authors declare that the research was conducted in the absence of any commercial or financial relationships that could be construed as a potential conflict of interest.
